# Update on the Role of β2AR and TRPV1 in Respiratory Diseases

**DOI:** 10.3390/ijms251910234

**Published:** 2024-09-24

**Authors:** Sara Manti, Antonella Gambadauro, Francesca Galletta, Paolo Ruggeri, Giovanni Piedimonte

**Affiliations:** 1 Pediatric Unit, Department of Human Pathology in Adult and Developmental Age “Gaetano Barresi”, University of Messina, Via Consolare Valeria 1, 98124 Messina, Italy; smanti@unime.it (S.M.); francygall.92@gmail.com (F.G.); 2 Pulmonology Unit, Department of Biomedical and Dental Sciences, University of Messina, Via Consolare Valeria 1, 98124 Messina, Italy; 3 Office for Research and Departments of Pediatrics, Biochemistry, and Molecular Biology, Tulane University, New Orleans, LA 70112, USA; gpiedimonte@tulane.edu

**Keywords:** asthma, bronchiolitis, β2AR, cystic fibrosis, COPD, respiratory diseases, RSV, TRPV1

## Abstract

Respiratory diseases (RDs) constitute a common public health problem both in industrialized and developing countries. The comprehension of the pathophysiological mechanisms underlying these conditions and the development of new therapeutic strategies are critical for improving the quality of life of affected patients. β2-adrenergic receptor (β2AR) and transient receptor potential vanilloid 1 (TRPV1) are both involved in physiological responses in the airways. β2AR is implicated in bronchodilation, mucociliary clearance, and anti-inflammatory effects, while TRPV1 is involved in the mediation of pain and cough reflexes. In RDs, such as respiratory infections, asthma, chronic obstructive pulmonary disease (COPD), and cystic fibrosis, the concentration and expression of these receptors can be altered, leading to significant consequences. In this review, we provided an update on the literature about the role of β2AR and TRPV1 in these conditions. We reported how the diminished or defective expression of β2AR during viral infections or prolonged therapy with β2-agonists can increase the severity of these pathologies and impact the prognosis. Conversely, the role of TRPV1 was pivotal in neuroinflammation, and its modulation could lead to innovative treatment strategies in specific patients. We indicate future perspectives and potential personalized treatments in RDs through a comprehensive analysis of the roles of these receptors in the physiological and pathological mechanisms of these pathologies.

## 1. Introduction

Respiratory diseases (RDs) are conditions affecting the lungs and the airways. RDs constitute a challenge to the public health systems in industrialized and developing countries due to their frequency and economic burden. In 2019, chronic RDs affected 454.6 million people and represented the third main cause of mortality globally [1]. Asthma and chronic obstructive pulmonary disease (COPD) are the principal culprits of chronic RDs, and numerous risk factors (e.g., smoking, air pollution, and high body mass index) are linked to these conditions [1]. 

RDs involve several cellular structures, from airway epithelial cells to immune cells. Their therapeutic management differs according to the specific pathophysiology of each disease. Membrane proteins and ion channels have a key role in their respiratory physiology and pathophysiology [2]. β2-adrenergic receptor (β2AR) is extensively expressed in the upper and lower respiratory tract, where it is responsible for several functions, such as bronchodilation, vasodilatation, mucociliary clearance, and anti-inflammatory actions [3]. Transient receptor potential vanilloid 1 (TRPV1) is prominently expressed in sensory nerves and non-neurological sites, such as the airway epithelium, where it is involved in pain sensation and inflammatory responses. 

The purpose of this review was to explore the current understanding of the contributions of β2AR and TRPV1 in lung pathophysiology. We further discuss potential areas for future research to better assess the impact of these transmembrane proteins in RDs. By combining the key terms “TRPV1” OR “Transient receptor potential vanilloid 1” OR “β2AR” OR “β2-adrenergic receptor” AND “respiratory diseases” OR “asthma” OR “wheezing” OR “respiratory infections” OR “COPD” OR “cystic fibrosis” in a computerized search of PubMed, we provide a complete overview of the current literature, based on a critical evaluation without standardized methodologies or statistical analyses.

## 2. β2AR and TRPV1: Structure and Function

### 2.1. β2AR

βARs are transmembrane glycoprotein structures belonging to the superfamily of G protein-coupled receptors (GPCRs) [4,5]. βARs are divided into three subtypes: β1ARs are mostly distributed on cardiomyocytes; β2ARs are mainly expressed on airway smooth muscles (ASMs), cardiomyocytes, uterine muscles, alveolar type I and II cells, mast cells, mucous glands, epithelial cells, vascular endothelium, eosinophils, lymphocytes, and skeletal muscles; β3ARs are principally involved in lipid metabolism in the adipose tissue [6,7,8,9]. Concerning β2ARs, their distribution in human lungs is prevalent in distal airways, especially in the alveolar walls [10,11]. The gene encoding for β2AR (*ADRβ2*) (MIM#109690) is located on chromosome 5q32 and contains only one exon [12]. This receptor mediates several physiological airway functions, including bronchodilation, vasodilatation, mucociliary clearance, and numerous anti-inflammatory effects (e.g., mast cell stabilization and cytokine production) [3]. 

**Figure 1:**
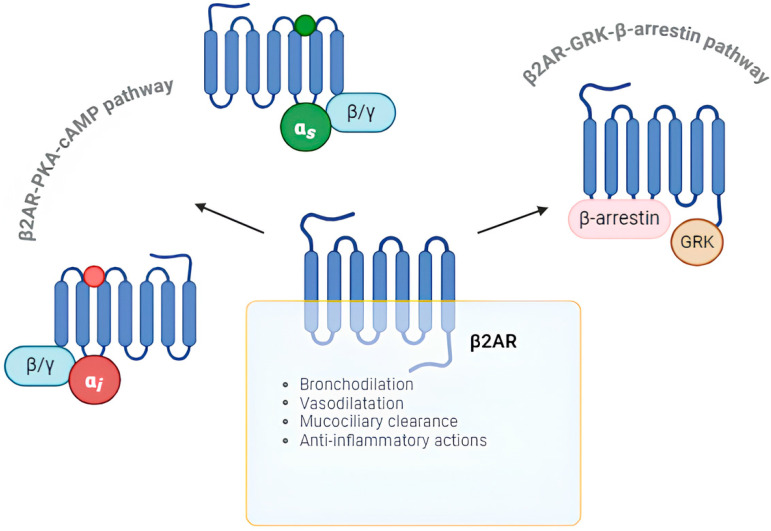
β2AR is a transmembrane glycoprotein belonging to the superfamily of G protein-coupled receptors (GPCRs) [4,13]. Most of the functions of this receptor are mediated by two pivotal pathways: the β2AR-PKA-cAMP pathway and the β2AR-GRK-β-arrestin pathway. The first one requires the participation of stimulatory or inhibitory G protein (α, β, and γ subunits) [15]. The stimulatory α-subunit activates the adenylyl cyclase (AC), which converts adenosine triphosphate (ATP) into cyclic adenosine monophosphate (cAMP) [7]. In the second pathway, intracellular cAMP levels activate the G protein-coupled receptor kinase (GRK), which phosphorylates the receptor, leading to the recruitment and coupling of β-arrestin [24]. β-arrestin participates in the desensitization, internalization, and degradation of β2AR [25,26]. The main physiological airway activities of β2AR include bronchodilation, vasodilatation, mucociliary clearance, and anti-inflammatory actions (e.g., mast cell stabilization and cytokine production) [3,17,22,23].

The structure of β2AR consists of a polypeptide chain with seven transmembrane α-helix regions and an eighth helix arranged parallel to the cytoplasmatic surface of the plasma membrane [13] (Figure 1). There are three extracellular loops, with an amino (N)-terminus, and three intracellular loops, with a carboxy (C)-terminus with serine and threonine phosphorylation sites [14]. Most of the functions of the transmembrane β2-receptors are mediated by stimulatory G (Gs)-protein, which consists of α, β, and γ subunits [15]. β2AR exists in two different states, activated and inactivated, which are in balance. This receptor is activated by binding catecholamines (e.g., epinephrine) or other agonists. The ligand binding induces the receptor coupling with inactive guanosine diphosphate (GDP)-bound G protein complex and a subsequent guanosine triphosphate (GTP) exchange activates the G proteins-receptor complex [16]. The activation is provided by the binding of the α-subunit of the Gs protein to a molecule of GTP; conversely, it is inactivated when GTP is replaced by GDP, inducing dissociation of the α-subunit of Gs. The α-subunit activates the enzyme adenylyl cyclase (AC), which converts adenosine triphosphate (ATP) into cyclic adenosine monophosphate (cAMP) [7]. In the airways, cAMP induces ASM relaxation by both activating protein kinase A (PKA), which phosphorylates various regulatory proteins involved in the muscle tone and reducing intracellular calcium ion (Ca^2+^) concentration, which leads to the relaxation of ASM [7]. The role of PKA in inducing ASM relaxation has never been straightforwardly demonstrated, but it is assumed to lead to either a reduction in intracellular Ca^2+^ concentration or lower Ca^2+^ cellular sensitivity, which both impair ASM contraction [17]. β2-agonists and other cAMP-raising agents modulate Ca^2+^ intracellular homeostasis in ASM, resulting in a dynamic process that alternates between Ca^2+^ influx and efflux. A key element of this homeostasis is the Ca^2+^ removal from the cytosol, which is mainly facilitated by the sarco/endoplasmic reticulum Ca^2+^-ATPase (SERCA) transporter. The uptake of intracellular Ca^2+^ through the SERCA transporters facilitates ASM relaxation. Thus, bronchodilation results from cAMP accumulation after the β2AR activation [17]. The stimulation of cholinergic muscarinic receptors, coupled with inhibitory G (Gi)- protein, decreases the cAMP levels, leading to ASM contraction and bronchoconstriction [18]. ASM relaxation can also be induced by cAMP-independent mechanisms involving the direct interaction between Gs and potassium ion (K^+^) channels [19]. Of these channels, the most critical seem to be the big-conductance Ca^2+^-activated K^+^ channels (BK_Ca_), which are abundant in ASM and a substrate for PKA [20,21]. Beyond its role in ASMs, the β2AR-PKA-cAMP pathway can interfere with the airway epithelial cells by accelerating mucociliary clearance and the polymorphonuclear leukocytes (especially eosinophils and neutrophils) by increasing intracellular cAMP levels and attenuating oxidative burst, resulting in anti-inflammatory effects [22,23]. 

The balance between activated and inactivated forms of β2ARs depends on intracellular cAMP levels. The cAMP accumulation stimulates the phosphorylation of the C-terminus of β2ARs by G protein-coupled receptor kinase (GRK), leading to the dissociation of the α-subunit of the Gs protein and the recruitment and coupling of β-arrestins [24]. β-arrestins are scaffold proteins that contribute to the desensitization, internalization, and degradation of β2ARs, mediating diverse signaling pathways, including extracellular signal-regulated kinase (ERK), p38 mitogen-activated protein kinase (MAPK), and nuclear factor-kappa B (NF-kB) [25,26]. By activating the ERK and p38 MAPK pathways, these proteins can induce the following: 1. the transcription and expression of the mucin-5AC (MUC5AC) gene, increasing the airway mucus secretion; 2. the mitosis of ASM cells; 3. the expression of chemokines in eosinophils [26,27]. Furthermore, β-arrestins can modulate the activation of the NF-kB pathway, which regulates the expression of chemokines and the polarization of macrophages [24,28]. Thus, the β2AR-GRK-β-arrestin pathway is implicated in inflammatory responses in the airways. 

The two kinases, GRK and PKA, phosphorylate the activated form of β2AR, inducing its desensitization. The desensitized β2AR is then internalized into endosomes, dephosphorylated, and reprocessed to the plasma membrane as a resensitized form, a process that is facilitated by the activity of a specific protein phosphatase [29,30].

### 2.2. TRPV1

TRPV1 is a Ca^2+^-permeable, non-selective cation channel included in the TRPV subfamily (TRPV1–6), which is part of the TRP ion channel family, widely distributed in the cellular membranes in humans and mammals [31]. TRPV1 is expressed in sensory nerves, and it is mainly located in non-myelinated C-fibers and a small subset of thin myelinated Aδ fibers. TRPV1-positive neural fibers innervate the respiratory system, from the upper (nose, larynx, and trachea) to lower airways (alveoli, ASM, and blood vessels) [32,33]. TRPV1 density is highest in the upper airway and gradually declines in the lower tracts [34]. Furthermore, this receptor is expressed in specific sites (e.g., vascular endothelium, respiratory system, skin, and muscle) and cellular groups (e.g., inflammatory and immune cells and adipocytes) [33]. The gene encoding for this receptor (*TRPV1*) (MIM#602076) is located on chromosome 17p13.2 and contains nineteen exons [35].

**Figure 2:**
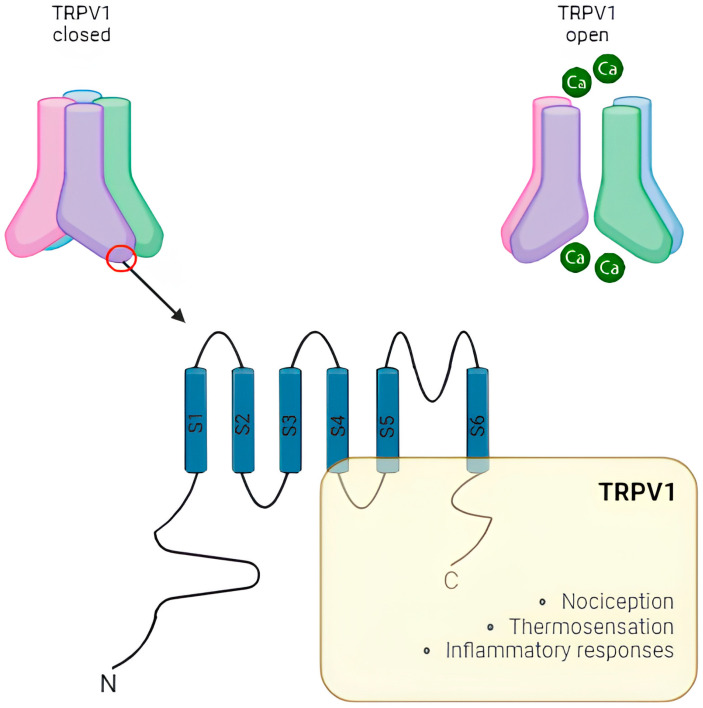
TRPV1 is a Ca^2+^-permeable channel belonging to the TRP ion channel family [31]. It is a multimeric protein with a four-fold symmetric axis forming a central ion channel surrounded by protein subunits. Every subunit is constituted by an N-terminus, a transmembrane domain formed by six transmembrane helices (S1–S6), and a C-terminus [36]. This channel can be stimulated by various endogenous and exogenous stimuli, including capsaicin, elevated temperature (>43 °C), protons, pro-inflammatory cytokines, and toxins (e.g., lipopolysaccharide) [37,38]. It participates in nociception, thermosensation and inflammatory response [33,34,40].

Structurally, TRPV1 is a multimeric protein with a four-fold symmetry axis forming a central ion channel enclosed by protein subunits. Every subunit has an N-terminus (with six ankyrin repeat domains), a transmembrane domain formed by six transmembrane helices (S1-S6), and a C-terminus (Figure 2). The S1-S6 transmembrane helices represent voltage-sensor-like domains, and at the N-terminus, there are ATP- and calmodulin-binding sites [36]. This channel can be stimulated by various endogenous and exogenous stimuli, including capsaicin, elevated temperature (>43 °C), protons, pro-inflammatory cytokines, and toxins (e.g., lipopolysaccharide) [37,38]. Once activated, a rearrangement takes place in the S5-S6 outside the pore-loop region of the central ion channel, while the external voltage-sensor-like domains S1-S6 remain unchanged [39,40]. Capsaicin causes burning pain by selectively activating sensory nerves. The capsaicin-binding site is intracellular on TRPV1 receptors, and the interaction induces the influx of Ca^2+^ and sodium ions (Na+), depolarizing the cell [40]. Moreover, TRPV1 is also a sensor of high temperature and acidic extracellular pH (i.e., protons). The reaching of the transition temperature (i.e., ~42 °C) induces the channel opening thanks to the thermosensitive property localized in the S1-S4 domains [41,42]. 

The aminoacidic structure of TRPV1 contains many phosphorylation sites for protein kinase C (PKC), PKA, and Ca^2+^/calmodulin-dependent protein kinase II (CaMKII), implicating regulatory modulation by these kinases [43,44,45]. Direct phosphorylation of TRPV1 at specific intracellular sites by PKA and PKC increases the receptor sensitivity [46,47]. However, the specific phosphorylation by PKA of residues Ser116 and Thr370 in the N-terminus is associated with desensitization [45]. Thus, the balance between phosphorylation and dephosphorylation is pivotal for TRPV1 activity [48]. Phospholipase C (PLC) influences TRPV1 activity by hydrolyzing the membrane phospholipid phosphatidylinositol 4,5-bisphosphate (PIP2) into diacylglycerol (DAG) and inositol 1,4,5-trisphosphate (IP3). When PIP2 is coupled to TRPV1, it promotes receptor inhibition, while its hydrolysis induces receptor activation. Both IP3 and DAG stimulate PKC, increasing TRPV1 phosphorylation and activation [46,49].

## 3. β2AR and TRPV1 in Respiratory Diseases

### 3.1. Respiratory Infections

Respiratory infections are a common public health problem, especially in infants and children [50,51]. They are typically caused by seasonal viruses, mainly affecting the upper respiratory tract (URT) [52]. However, in infants and children, the involvement of the lower respiratory tract (LRT) is not rare and is frequently caused by *Respiratory Syncytial Virus* (RSV) and *Human Rhinovirus* (HRV) [53,54,55]. The impact of these two viruses on β2AR and TRPV1 has attracted attention, especially in patients with bronchiolitis. 

β2-agonists, such as salbutamol, are not recommended in bronchiolitis management because they do not improve oxygen saturation, duration of symptoms or length of stay in the hospital [56,57]. The reason for their lack of efficacy was determined in previous studies. Moore et al. reported that RSV infection reduced β2AR density in infected cells by 32% compared with the controls (mock-infected cells). Moreover, RSV attenuated isoproterenol (ISO)-induced cAMP formation in a time- and concentration-dependent manner. These two modifications on β2AR density and ISO-induced cAMP formation were mainly expressed in individuals homozygous for the Arg16Gln27 haplotype, suggesting a potentially different pattern severity of RSV infection in patients with specific haplotypes [58]. RSV infection may induce β2AR desensitization either directly by the G protein-coupled receptor kinase 2 (GRK2) or protein kinase C zeta type (PKCζ) phosphorylation of the receptor or indirectly by the sequestration of the α-subunit of Gs [59]. In a mouse model, the ligation of CXCR2 (or interleukin 8 receptor β, IL8Rβ) by the chemokine named keratinocyte cytokine (KC), the murine homolog of human CXCL8 (or IL8) released in response to RSV infection, induced the activation of GRK2 or PKCζ, with the subsequent phosphorylation and desensitization of β2AR [60]. Thus, these findings emphasized how the RSV infection may decrease the response to β2-agonists by reducing the expression and the sensitization of β2AR, blocking this receptor in a phosphorylated and desensitized form. More recently, Harford et al. confirmed the prevalence of β2AR phosphorylated form due to RSV infection [61]. However, they demonstrated that RSV simultaneously caused more effects, such as a proteasome-mediated cleavage and degradation of β2AR (which explains the low density of the receptor on the plasma membranes) and a β2AR ligand-independent activation of AC. This non-canonical activation of AC results in impaired cAMP synthesis and increased levels of intracellular Ca^2+^, generating a pro-contractile phenotype in RSV-infected ASM cells [61]. It could be supposed that RSV infection activates the cAMP-PKA axis in a non-canonical and ligand-independent way, leading to ASM contraction and bronchoconstriction. Furthermore, the non-responsivity to β2-agonists could be due to the β2AR’s low density and high rate of their desensitized forms in the RSV-infected airways. 

While β2AR density decreases in the airways during RSV infection, TRPV1 levels increase in the epithelial and neuronal cells [62,63]. Interestingly, the upregulation in TRPV1 expression seems independent of viral replication and related to virus-induced soluble factors, such as IL8 [62]. TRPV1 directly increases IL8 levels by activating the c-Jun N-terminal kinase 1 (JNK1)-dependent and JNK1-independent signaling pathways through the transforming growth factor-activated kinase 1 (TAK1) phosphorylation. The stimulation of these pathways generates NF-kB positive feedback control of JNK1/2 phosphorylation, which, in turn, increases the levels of IL8 [62]. This cytokine, along with other mediators, may facilitate the interaction of viral proteins with cell surface toll-like receptors (TLRs), enhancing the TRPV1 expression [62]. TRPV1 is implicated in regulating irritant-induced airway responses, particularly in cough [64]. During RSV infection, TRPV1 upregulation, together with the overexpression of nerve growth factor (NGF) and neurokinin 1 (NK1) receptor in the lungs, induces increased neuroinflammation, which may contribute to long-lasting airway inflammation and hyperreactivity [34,65,66]. In the acute phase, RSV stimulates the TRPV1-dependent Ca^2+^ influx in bronchial epithelial cells, which are responsible for augmented mucus production, disrupted barrier permeability, and enhanced bronchoconstriction, especially in asthmatic children [67]. 

In summary, during RSV infection, the functional impairment of β2AR and overexpression of TRPV1 facilitate bronchoconstriction and the persistence of inflammation in affected patients. Analyzing the cytokine profiles involved in regulating these receptors could be useful in identifying future target treatments. Interestingly, RSV enhances the expression of IL8, which is involved in β2AR desensitization and TRPV1 upregulation [60,62]. Authors showed the presence of specific haplotypes or genetic variants related to disease severity phenotypes [58,68]. In the future, a personalized approach could improve the management of this condition.

HRV infects airway epithelial cells by binding to ICAM-1 [69]. In vitro studies showed that β2-agonists (such as salbutamol and terbutaline) reduce ICAM-1 expression, suggesting their use could decrease HRV entry and spread in the airways [70,71]. Despite these promising results, Bochkov et al. reported that the pre-treatment with a combination of budesonide and formoterol 24 h before HRV incubation did not affect viral replication in bronchial epithelial cells [72]. Interestingly, HRV induces β2AR desensitization in human ASM cells, probably thanks to autocrine prostaglandin (PG) production [73]. Recent studies have shown how respiratory viral infections can reduce the efficacy of β2-agonists (e.g., salmeterol and formoterol) during asthma and COPD exacerbations thanks to the activation of PGE2, an eicosanoid produced by cyclooxygenase (COX) against infection [74,75]. This reduction of the medication function is related to β2AR desensitization in ASM cells due to the PGE2 interaction with the prostanoid E 2 (EP2) receptor [75]. Moreover, PGE2 produced in response to inflammation seems to increase nerve fiber sensitivity, TRPV1 activity, and cough stimulation [76]. It has been demonstrated that administering an EP3 receptor antagonist inhibits PGE2-induced cough in a guinea pig model [76]. The use of selective EP2 or EP3 antagonists may offer a potential treatment option for respiratory infectious diseases in the future, especially in patients with asthma and COPD.

### 3.2. Asthma

Asthma is a complex and heterogeneous disease characterized by chronic inflammation of the airways [77]. The development of asthma is multifactorial, and although the causal mechanisms are not entirely understood, it is well-known that genetic factors, atopy, and early respiratory infections play a pivotal role in its pathogenesis [78,79].

Early-life or prenatal respiratory viral infections, specifically HRV and RSV, are associated with asthma onset [80,81]. Sigurs et al. demonstrated that 32% of infants hospitalized with severe RSV infection subsequently developed allergen sensitization by 3 years of age, compared with only 9% of age- and sex-matched controls [80]. A study on a three-dimensional human lung organoid (HLO) model showed how prenatal RSV infection may modify the architecture and cytological profile of the fetal lung by reducing the population of ciliated cells, increasing the proliferation of reactive club cells, and inducing the disruption of the actin cytoskeleton. Moreover, RSV also changes the expression of key receptors, inducing the presence of higher levels of phosphorylated β2AR and TRPV1 in the mesenchymal and epithelial compartments [63]. These modifications increase the predisposition to developing airway inflammation and hyperreactivity, both present in wheezing and asthma. Bronchial hyperreactivity may also be the consequence of the development of a pro-contractile phenotype related to increased cytosolic free Ca^2+^ in children with viral wheezing [61] and asthma [82]. The overexpression of TRPV1 channels and the intracellular Ca^2+^ levels in the bronchial epithelium are higher in asthmatic children compared with age-matched healthy controls and asthmatic adults [82]. Thus, viral infection affects TRPV1 expression regardless of the asthma status in children but not in adults [67,82]. 

The role of *TRPV1* and *ADRβ2* genetic variants was widely studied in past research, but contrasting results in different populations have been obtained. A meta-analysis of case-control studies, including 46 studies, analyzing the association of *ADRβ2* polymorphisms and the risk of asthma identified four single-nucleotide polymorphisms (SNPs): Arg16Gly (A46G, rs1042713), Gln27Glu (C79G, rs1042714), Thr164Ile (C491T, rs1800888), and Arg19Cys (T-47C, rs1042711) [83]. Significant correlation was found for Arg16Gly polymorphism in the South American population in an analysis stratified by ethnicity via dominant model comparison (OR = 1.754, 95% CI = 1.179–2.609, I2 = 16.9%, studies = 2, case = 314, control = 237). A protective association has been reported for the Gln27Glu polymorphism in children via recessive model comparison (OR = 0.566, 95% CI = 0.417–0.769, I2 = 0.0%) and homozygote genotype comparison (OR = 0.610, 95% CI = 0.434–0.856, I2 = 0.0%) and in adults via dominant model comparison (OR = 0.864, 95% CI = 0.768–0.971, I2 = 46.9%) [83]. More recently, a specific genotype (AA) of the Arg16Gly variant was associated with increased rates of hospitalization in asthmatic children and adolescents compared with other genotypes (AG and GG) [84]. Cantero-Recasens et al. first described the involvement of TRPV1 in asthma pathophysiology and reported a protective effect of the TRPV1-Val-585 variant against the presence of wheezing or cough among asthmatics [85]. Further studies are needed to better define the implication of these SNPs on asthma severity and to identify the protective or non-protective effects of these variants. The results of future research could improve the personalization of treatment strategy. 

β2-agonists and corticosteroids are the main treatments used in managing asthmatic patients from childhood to adulthood [77]. Despite the central role of β2-agonists in asthma treatment, data suggest that an increased prescription of this medication is related to higher asthma mortality [86,87,88]. Chronic use of β2-agonists induces tachyphylaxis and a rebound in airway hyperresponsiveness [89]. Approximately 70% of asthmatic patients with chronic use of β2-agonists lose the ability of this medication to induce bronchodilation [90,91]. The β2AR dysfunction in asthmatic human ASM cells is the result of the reduced resensitization of the receptor characterized by reduced dephosphorylation of β2AR due to diminished endosomal protein phosphatase 2A (PP2A) activity. The reduced PP2A activity, which normally resensitizes the receptor, permitting its recycling to the plasma membrane, induces the endosomal accumulation of phosphorylated β2AR, which is the desensitized form of the receptor. Furthermore, high levels of phosphodiesterase-4 (PDE4) in asthmatic patients accelerate the catalysis of cAMP and contribute to loss in β2AR response [92]. SNPs in *ADRβ2*, such as Arg16Gly (rs1042713), Gln27Glu (rs1042714), and Arg19Cys (rs1042711), may be associated with the downregulation of β2AR. In turn, the Thr164Ile (rs180088) polymorphism reduces the receptor binding affinity to β2-agonists [84,93,94]. Conversely, corticosteroids increase the number of β2ARs in human lungs in a time- and dose-dependent manner and prevent the β2-agonists-induced downregulation of β2ARs [95,96,97]. The synergism is bidirectional: β2AR action improves the anti-inflammatory activity of corticosteroids by enhancing the translocation of the glucocorticoid receptor (GR) from the cytoplasm to the cellular nucleus [98]. The effect of this synergism is the possibility of using low-dose drug combination therapy, minimizing the side effects of the drugs.

The expression of TRPV1 is increased in the airway epithelium of asthmatic patients and is more conspicuous in those with severe and uncontrolled disease [99]. The TRPV1 functions and the effects of TRPV1 antagonism on airway inflammation gained increasing interest and attention in the literature. Rehman et al. reported that the inhibition of TRPV1 reduced airway hyperactivity and remodeling, goblet cell metaplasia and subepithelial fibrosis in IL13-induced asthma model in BALB/c mice [100]. In another study in BALB/c mice, the sample group sensitized with ovalbumin (OVA) showed increased airway hyperactivity and the development of an asthmatic lung phenotype compared with controls. In this OVA challenge group, the expression of the TRPV1 receptor and the release of type 2 (T2) cytokines (IL3, IL5, IL5) were significantly elevated. The inhalation of TRPV1 antagomir, a small interfering mRNA (siRNA) directed toward TRPV1, efficiently controlled eosinophilic airway inflammation and remodeling. TRPV1 antagomir suppressed epithelial-derived cytokines, such as thymic stromal lymphopoietin (TSLP), IL33, and IL25, which are important regulators of T2 cytokines-associated inflammation [101]. Since TRPV1 expression seems to be related to asthma severity, its suppression could be helpful in decreasing chronic airway epithelial injury and asthma features, as observed in animal models. TRPV1 expressed in sensory neurons innervating the airways can be stimulated by inhaled irritants that increase Ca^2+^ influx and release of neuroinflammatory substances. Of these substances, pro-inflammatory neuropeptides, such as calcitonin gene-related peptide (CGRP) and NK1, are responsible for the development of neurogenic inflammation in the airways [102,103,104]. These neuropeptides lead to bronchoconstriction, mucus hypersecretion, airway irritation, and cough and influence the recruitment of neutrophils and eosinophils in the lungs, all elements associated with asthma [2,103]. Moreover, the activation of TRPV1 in bronchial epithelial cells promotes the release of pro-inflammatory mediators, such as ILs, PGE2, NGF, and tumor necrosis factor α (TNFα), sustaining airway inflammation and airway hypersensitivity [105,106,107]. TRPV1-positive sensory neurons expressed the protease-activated receptor 2 (PAR2), which is implicated in neurogenic inflammation and hyperalgesia [108,109]. In the airways, PAR2 activation is associated with inflammatory responses, including exaggeration of allergic reactions, bronchoconstriction and plasma protein extravasation [110,111]. PAR2 stimulation upregulates the function of TRPV1 via a PKC-dependent mechanism, regulating the TRPV1 activity during neurogenic inflammation in the airway by the concomitant release of arachidonic acid and PGE2 [112,113]. Furthermore, in a study in primary human nasal epithelial cells and murine tracheal epithelial cells, both TRPV1 and PAR2 were implicated in innate responses against airborne allergens (i.e., house dust mite and *Alternaria alternata*) by an allergen-induced ATP release promoting IL33 secretion [114]. These data suggest that TRPV1 is strictly related to asthma pathogenesis and might be a promising therapeutic target, especially in patients with severe and uncontrolled asthma.

### 3.3. COPD

COPD is a heterogeneous lung disease characterized by chronic respiratory symptoms due to defects in the airways, which cause chronic, often progressive, airflow obstruction. This condition results from genetic and environmental factors (e.g., cigarette smoking) occurring during an individual’s lifetime [115]. 

Several studies have supported that *ADRβ2* polymorphisms may influence the COPD severity and the sensitivity to the treatment. People homozygous for Arg16 have an increased risk of COPD and asthma, and the Arg16/Gln27 haplotype is associated with more severe respiratory symptoms in middle-aged and older adults with COPD [116]. The genotypes Gly16Arg (rs1042713) and Gln27Glu (rs1042714) increase the risk of severe exacerbation in COPD, potentially mainly by genetic influence of the 16Arg allele in rs1042713 [117]. In a randomized, double-blind study, COPD patients with the Arg16Arg genotype reported lower exacerbation risk compared with Arg16Gly and Gly16Gly genotypes in the salmeterol group, highlighting a different treatment response [118]. Identifying these polymorphisms could be used in future personalized treatment strategies to identify patients more at risk of severe disease.

Cigarette smoking is one of the main causative agents of COPD. Smoke exposure produces an early respiratory inflammation mediated by neuropeptides (such as NK1) that increases the recruitment of eosinophils and neutrophils and the production of reactive oxygen species (ROS) [119,120]. The principal ROS are hydrogen peroxide (H2O2) and hydroxyl radical (·OH) [121]. ROS directly activate TRPV1 receptors, stimulating the vagal lung afferent fibers and inducing excessive cough in smokers with COPD [122,123]. Furthermore, smoke exposure can modify the lung phenotype of TRPV1 receptors, increasing their expression and functionality. Thus, this phenotypic switch justifies the excessive cough responses to a range of inhaled irritants in smokers [123]. Apart from the smoking status, patients with COPD have a lower threshold to cough by different stimuli, including capsaicin [124,125]. In an in vivo study, an inhibitor (AMG9810) of TRPV1 diminished oxidative stress, prevented Ca+ influx, inhibited inflammatory response and improved antioxidant gene expression in bronchial and alveolar epithelial cells exposed to cigarette smoking [126]. Furthermore, the administration of a TRPV1 antagonist (SB-705498) significantly improved cough frequencies in patients with chronic cough [127]. However, researchers are still evaluating the role of TRPV1 inhibitors as a potential therapy for COPD, and it could represent a future therapeutic challenge in the management of these patients. Moreover, recent studies have analyzed the role of anti-oxidative treatments in modulating TRPV1 expression in COPD. A study in cigarette smoking-challenged mice exhibited how total flavonoids, the leading active components derived from loquat leaves, inhibited inflammation and oxidative stress response through down-regulating TRPV1 expression. This anti-oxidative treatment also reduced the synthesis of IL6, IL1β, TNFα, and nitric oxide (NO), modulating the neurogenic inflammation and oxidative stress observed in chronic RDs [128]. The use of antioxidants with TRPV1 down-regulation properties could improve COPD management in future studies.

### 3.4. Cystic Fibrosis

Cystic fibrosis is a hereditary disease defined by an altered homeostasis of ion and water transports in secretory epithelial surfaces, including the airways. The disease is caused by mutations in the *cystic fibrosis transmembrane conductance regulator* (CFTR) gene coding for an epithelial chloride channel, which leads to impaired mucociliary clearance [129,130]. Although a wide range of literature research has analyzed the role of CFTR, few studies have defined the importance of other receptors in the interaction with CFTR and their possible application in disease management. 

There are several causes of cough in cystic fibrosis, such as airway inflammation and impairment in mucociliary clearance. The increased cough frequency is one of the main signs of pulmonary exacerbations in patients with cystic fibrosis [131]. However, a comprehensive evaluation of the cough sensitivity in this condition is modest. In 1997, Chang et al. showed a pivotal difference between children affected by cystic fibrosis and asthma or recurrent non-asthmatic cough. Cough sensitivity to capsaicin was lower in children with cystic fibrosis compared to asthmatic children and healthy controls. Furthermore, children with cystic fibrosis had an increased threshold to cough compared to controls [132]. A study on cough in children with cystic fibrosis did not find any statistically significant difference in cough frequency before and after treatment for pulmonary exacerbations despite significant improvements in spirometry, suggesting a possible reduced sensitivity in this age group [133]. In adults with cystic fibrosis, cough sensitivity has not been studied, but the cough rate in patients treated for exacerbations seems to decrease at the end of treatment, in contrast with what was observed in children [134]. Since the capsaicin receptor is TRPV1, it could be supposed that a different pattern of activation of this receptor in the lung sensory neurons of patients with cystic fibrosis is present, with a low range of cough sensitivity in children and not in adults. However, further studies are needed to confirm this supposition. Interestingly, CFTR and TRPV1 have structural similarities in the cytoplasmatic domains, and both channels are regulated by ATP. It was demonstrated that capsaicin—by binding to the cytoplasmatic domain of CFTR—improves the activity of wild-type and mutant CFTR channels [135]. This result highlights the importance of future studies on TRPV1-CFTR interactions to develop future therapeutic strategies in patients with cystic fibrosis.

β2-agonists are commonly used in the management of patients with cystic fibrosis. CFTR can be activated by β2AR via raising cAMP intracellular levels and mediating PKA activation. In the airways, the β2AR-CFTR interaction is facilitated by scaffold proteins, such as Na+/H+ Exchanger Regulatory Factor (NHERF1), permitting its interaction with PKA and stabilizing it on the plasma membrane [136,137]. Studies in vitro human or in vivo animal models showed that single doses of β2-agonists increase mucociliary clearance and bronchodilation, explaining the frequent use of short-acting β2-agonists (such as albuterol) in clinical practice for daily airway clearance regimens in cystic fibrosis [138,139,140]. However, chronic exposure to β2-agonists (such as albuterol and formoterol) reduces cAMP generation following direct AC stimulation and PDE inhibition, which also leads to a defective CFTR activation. The reduction in CFTR function following long-term β2-agonists use seems to have little consequences in healthy individuals, and it appears to be more significant in cystic fibrosis, especially on modulatory therapy such as pharmacologic F508del correction [141]. Patients with cystic fibrosis may show different responses to β2-agonists. Some studies revealed individual differences related to specific *ADRβ2* polymorphisms, and the GG haplotype was associated with a greater response to albuterol compared with other haplotypes. However, there is no evidence of the impact of these polymorphisms on long-term outcomes in this population, and future studies could resolve this issue and improve the management of these patients [142].

## 4. Conclusions

As we showed in this review, β2AR and TRPV1 are widely involved in physiological mechanisms in the upper and lower airways, and their role in the pathogenesis of RDs is also crucial. To our knowledge, this is the first review summarizing the functions and dysfunctions of these two receptors in the main RDs. Previous studies only analyzed the individual receptor without reporting the peculiar interplay between β2AR and TRPV1 in the development and persistence of RDs. In Table 1, we reported the key points of our analysis. Respiratory viral infections, such as RSV and HRV infections, can alter receptor expression and function, leading to bronchoconstriction and persistent airway inflammation. Moreover, viral infections occurring during early childhood are related to asthma onset, and genetic variants in *ADRβ2* and *TRPV1* can influence disease severity and patient response to treatment. Chronic use of β2-agonists can induce tachyphylaxis and decrease treatment effectiveness due to β2AR desensitization. In COPD, *ADRβ2* polymorphisms can be related to disease severity and progression, and future personalized therapeutic approaches may improve the management of this condition. Moreover, patients with COPD have a lower threshold to cough, which is justified by an increased response to different stimuli, especially smoke, which is responsible for the high expression of TRPV1 in the airways. In cystic fibrosis, TRPV1 activation could improve CFTR function and cough effectiveness, while β2AR stimulation increases mucociliary clearance and favors bronchodilation.

The understanding of the regulation of β2AR and TRPV1 and their interaction can be crucial for developing more effective and personalized treatments in RDs. In this review, we discussed the implication of receptor dysfunction in each pathology and reported the most relevant novelties of therapeutic strategies. Future research on receptor modulation and targeted signaling pathways could be helpful in developing new management strategies and improving the outcomes of these conditions.

## Figures and Tables

**Table 1:** Key points of the roles of β2AR and TRPV1 in respiratory diseases.

Respiratory Disease	β2AR	TRPV1
Respiratory infections	RSV infection reduces β2AR density in infected cells and attenuates ISO-induced cAMP formation [58]. RSV induces β2AR desensitization either directly by the GRK2 or PKCζ phosphorylation of the receptor or indirectly by the sequestration of the α-subunit of Gs [59,61]. IL8, released in response to RSV infection, induces the activation of GRK2 or PKCζ, with the subsequent phosphorylation and desensitization of β2AR [60]. RSV induces a proteasome-mediated cleavage and degradation of β2AR (reducing its density) and leads to impaired cAMP synthesis and increased levels of intracellular Ca^2+^, generating a pro-contractile phenotype in RSV-infected ASM cells [61]. HRV induces β2AR desensitization in human ASM cells, probably thanks to autocrine PG production [73]. Respiratory viral infections induce β2AR desensitization in ASM cells due to the PGE2 interaction with the EP2 receptor [75], reducing the efficacy of β2-agonists during asthma and COPD infectious exacerbations [74,75].	RSV increases TRPV1 expression in the epithelial and neuronal cells [62,63]. The upregulation in TRPV1 expression seems related to a NF-kB positive feedback control of JNK1/2 phosphorylation which increases IL8 levels, enhancing TRPV1 expression [62]. During RSV infection, TRPV1 upregulation, together with the overexpression of NGF and NK1 receptors in the lungs, induces increased neuroinflammation, which may contribute to long-lasting airway inflammation and hyperreactivity [34,65,66]. RSV stimulates the TRPV1-dependent Ca^2+^ influx in bronchial epithelial cells, which are responsible for increased mucus production, disrupted barrier permeability, and enhanced bronchoconstriction, especially in asthmatic children [67]. PGE2 produced during respiratory viral infections seems to increase TRPV1 activity and cough stimulation [76].
Asthma	Some *ADRβ2* polymorphisms are related to an increased risk of asthma, such as Arg16Gly polymorphism in the South American population. A protective association was reported for the Gln27Glu polymorphism [83]. The β2AR dysfunction in asthmatic human ASM cells is the result of the reduced resensitization of the receptor characterized by diminished dephosphorylation of β2AR due to decreased endosomal PP2A activity. Furthermore, high levels of PDE4 in asthmatic patients accelerate the catalysis of cAMP and contribute to loss in β2AR response [92]. Corticosteroids increase the number of β2ARs in human lungs in a time- and dose-dependent manner and prevent the β2-agonists-induced downregulation of β2ARs [95,96,97].	TRPV1-Val-585 variant seems to have a protective effect against the presence of wheezing or cough among asthmatics [85]. The expression of TRPV1 is increased in the airway epithelium of asthmatic patients and is more prominent in those with severe and uncontrolled disease [99]. In animal models, the inhibition of TRPV1 reduced airway hyperactivity and remodeling, goblet cell metaplasia and subepithelial fibrosis [100]. The activation of TRPV1 in bronchial epithelial cells promotes the release of pro-inflammatory mediators, such as ILs, PGE2, NGF, and TNFα, sustaining airway inflammation and airway hypersensitivity [105,106,107]. TRPV1-positive sensory neurons expressed the PAR2, which is implicated in neurogenic inflammation and hyperalgesia [108,109]. In the airways, PAR2 activation is associated with inflammatory responses, including exaggeration of allergic reactions, bronchoconstriction and plasma protein extravasation [110,111]. Both TRPV1 and PAR2 seems to be implicated in innate responses against airborne allergens [114].
COPD	Some ADRβ2 polymorphisms are associated with COPD severity. People homozygous for Arg16 have an increased risk of COPD, and the Arg16/Gln27 haplotype is associated with more severe respiratory symptoms in middle-aged and older adults with COPD [116]. The genotypes Gly16Arg (rs1042713) and Gln27Glu (rs1042714) increase the risk of severe exacerbation in COPD [117].	Smoke exposure can modify the lung phenotype of TRPV1 receptors, increasing their expression and functionality. This phenotypic switch justifies the excessive cough responses to a range of inhaled irritants in smokers [123]. Patients with COPD have a lower threshold to cough by different stimuli, including capsaicin [124,125].
Cystic fibrosis	CFTR can be activated by β2AR via raising cAMP intracellular levels and mediating PKA activation [136,137]. Single doses of β2-agonists increase mucociliary clearance and bronchodilation, explaining the frequent use of short-acting β2-agonists in clinical practice for daily airway clearance regimens in cystic fibrosis [138,139,140]. Chronic exposure to β2-agonists reduces cAMP generation following direct AC stimulation and PDE inhibition, which also leads to a defective CFTR activation. The reduction in CFTR function following long-term β2-agonists use seems to have little consequences in healthy individuals, and it appears to be more significant in cystic fibrosis, especially on modulatory therapy such as pharmacologic F508del correction [141].	Cough sensitivity to capsaicin (a well-known agonist of TRPV1) is lower in children with cystic fibrosis compared to asthmatic children and healthy controls. Furthermore, children with cystic fibrosis had an increased threshold to cough compared to controls [132].

β2AR = β2-adrenergic receptor; TRPV1 = transient receptor potential vanilloid 1; RSV = respiratory syncytial virus; ISO = isoproterenol; cAMP = cyclic adenosine monophosphate; GRK2 = G protein-coupled receptor kinase 2; PKCζ = Protein kinase C zeta type; IL8 = interleukin 8; Ca^2+^ = calcium ion; ASM = airway smooth muscle; HRV = human rhinovirus; PG = prostaglandin; EP2 = prostanoid E2; COPD = chronic obstructive pulmonary disease; NF-kB = nuclear factor-kappa B; JNK1/2 = c-Jun N-terminal kinase 1/2; NGF = nerve growth factor; NK1 = neurokinin 1; PP2A = protein phosphatase 2A; PDE4 = phosphodiesterase-4; TNFα = tumor necrosis factor α; PAR2 = protease-activated receptor 2; CFTR = cystic fibrosis transmembrane conductance regulator; PKA = protein kinase A.
